# Few-shot biomedical named entity recognition via knowledge-guided instance generation and prompt contrastive learning

**DOI:** 10.1093/bioinformatics/btad496

**Published:** 2023-08-07

**Authors:** Peng Chen, Jian Wang, Hongfei Lin, Di Zhao, Zhihao Yang

**Affiliations:** School of Computer Science and Technology, Dalian University of Technology, Dalian 116024, China; School of Computer Science and Technology, Dalian University of Technology, Dalian 116024, China; School of Computer Science and Technology, Dalian University of Technology, Dalian 116024, China; School of Computer Science and Engineering, Dalian Minzu University, Dalian 116600, China; School of Computer Science and Technology, Dalian University of Technology, Dalian 116024, China

## Abstract

**Motivation:**

Few-shot learning that can effectively perform named entity recognition in low-resource scenarios has raised growing attention, but it has not been widely studied yet in the biomedical field. In contrast to high-resource domains, biomedical named entity recognition (BioNER) often encounters limited human-labeled data in real-world scenarios, leading to poor generalization performance when training only a few labeled instances. Recent approaches either leverage cross-domain high-resource data or fine-tune the pre-trained masked language model using limited labeled samples to generate new synthetic data, which is easily stuck in domain shift problems or yields low-quality synthetic data. Therefore, in this article, we study a more realistic scenario, i.e. few-shot learning for BioNER.

**Results:**

Leveraging the domain knowledge graph, we propose knowledge-guided instance generation for few-shot BioNER, which generates diverse and novel entities based on similar semantic relations of neighbor nodes. In addition, by introducing question prompt, we cast BioNER as question-answering task and propose prompt contrastive learning to improve the robustness of the model by measuring the mutual information between query–answer pairs. Extensive experiments conducted on various few-shot settings show that the proposed framework achieves superior performance. Particularly, in a low-resource scenario with only 20 samples, our approach substantially outperforms recent state-of-the-art models on four benchmark datasets, achieving an average improvement of up to 7.1% *F*1.

**Availability and implementation:**

Our source code and data are available at https://github.com/cpmss521/KGPC.

## 1 Introduction

As a fundamental task in biomedical text mining, biomedical named entity recognition (BioNER) aims to locate and classify entity spans in a given sentence, which facilitates downstream tasks, such as relation extraction, event detection, and question answering (QA) (Yoon *et al.* 2022, Chen *et al.* 2022a, Wang *et al.* 2022b). However, current state-of-the-art (SoTA) models rely on large amounts of high-quality data manually annotated by domain experts, which are expensive and difficult to collect due to privacy and security restrictions, especially in medicine domains. Therefore, few-shot learning (FSL) is proposed to recognize unlabeled examples (query set) based on very few labeled samples (support set) and raising growing attention including in named entity recognition tasks, but it has not been extensively studied yet in the biomedical domain.

FSL involves characterizing different classes with few labeled samples. A line of research investigates how to allow a model to effectively classify unlabeled examples from the target domain by leveraging datasets in the rich-resource domains. Fritzler *et al.* (2019) explored few-shot named entity recognition with the Prototypical Network, which pre-trains on source domains and then perform word-level classification on target domains without training. Instead of learning category prototype, Yang and Katiyar (2020) calculated the nearest neighbor of each sample in query set. More recently, Chen *et al.* (2022b) proposed a novel generative framework with prompt-guided attention for few-shot named entity recognition. The core idea of these methods is based on similar textual patterns between the high-resource source domain and the low-resource target domain. However, cross-domain transfer learning inevitably brings domain shift problems.

**Figure 1.**
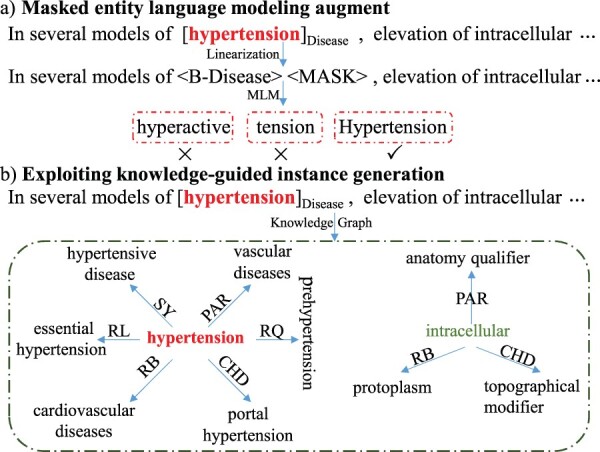
Illustrations of DA method for few-shot BioNER, (a) masked entity language modeling augment proposed by Zhou *et al.* (2022b), and we explore (b) exploiting knowledge-guided instance generation

Another line of research in few-shot named entity recognition considers data augmentation (DA). Different from sentence-level text augmentation, BioNER is a fine-grained token-level sequence labeling and suffers from the token-label misalignment issue when applying DA like back-translation (Sánchez-Cartagena *et al.* 2021). To explore DA on the few-shot named entity recognition, Dai and Adel (2020) proposed to randomly replace entity mentions using another mention with the same entity type from the original training set. However, random entity substitution operations may distort the original context and break the semantics. More recent research has resorted to the pre-trained masked language models (MLM) for DA in low-resource scenario (Ding *et al.* 2020, Zhou *et al.* 2022b). Given the labeled sentence with tokens being randomly masked, one can directly fine-tune MLM to generate new synthetic data without manual annotation. They avoided token-label misalignment issue but fine-tuning PLMs under low-resource scenario could result in over-fitting (Wang *et al.* 2022a). As a result, the predicted entity may be very similar to the original training instances or even generate incorrect entity mentions. As shown in Fig. 1a, after masking the named entity “hypertension” (B-Disease) in the sentence, the fine-tuning MLM predicts it as “hyperactive” or “tension,” which is obviously not a Disease entity.

Considering the biomedical knowledge graph contains abundant entities, we explore knowledge-guided instance generation for few-shot BioNER, which generates augmented data with diverse entities while avoiding the domain adaptive challenge from the source and target domain. As displayed in Fig. 1b, exploiting knowledge graphs from biomedical UMLS (https://www.nlm.nih.gov/research/umls/index.html), we can get the relations of candidate concepts “hypertension” and “intracellular.” To fit into the original context, only six types of relations are considered including “SY,” “RL,” “RB,” “RQ,” “PAR,” and “CHD” (refer to https://www.nlm.nih.gov/research/umls/knowledge_sources/metathesaurus/release/abbreviations.html for more detailed information on relation abbreviation description). Then corresponding candidate entities, such as “hypertension,” are replaced by their neighbor nodes and the entity types remain unchanged. Notably, we also replace the non-entity “intracellular” of the sentence. Thus, compared to MLM, knowledge-guided instance generation spawns more novel samples and ensures the quality of generation.

Nevertheless, an inevitable issue is that limited number of augmented examples may still cause a biased estimation of feature distribution in certain types, especially when there are 5, 20 or 50 labeled samples per class in the few-shot scenarios. To this end, we propose a novel prompt contrastive learning (CL) framework. Specifically, we introduce question prompts aiming to entity types and regard the entity mentions matching with the current question prompt as positive samples, while the unmatched as negative samples. Thus, we can (i) maximize the mutual information (MI) of matching query–entity pairs and (ii) minimize the MI of negative samples unmatched the query. In other words, we can punish the cross-entropy loss, by using the MI between the question prompt and the predicted entity, to construct a rectified feature that is less biased and more representative of the current question prompt. To verify the effectiveness of the model, we conduct extensive experiments on various few-shot settings. Particularly, in a low-resource scenario with only 20 samples, our approach obtains 63.5%, 60.5%, 61.4%, and 66.1% in *F*1 on four benchmarks, outperforming substantially recent SoTA models by 3.9–7.1 *F*1 score. To summarize, in this article, we study a more realistic scenario, i.e. FSL for BioNER. The contribution of this article is as follows:

- Based on the semantic relations of the knowledge graph, we propose knowledge-guided instances generation for few-shot BioNER, which enriches training samples for better generalization and is superior to recent DA by MLM.
- Introducing question prompts, we formulate BioNER as a QA task and propose prompt CL to improve the robustness of the model by measuring the MI between query and entity.
- Extensive experiments are conducted on four benchmark datasets under various few-shot settings. Particularly, in the 20-shot scenario, the results outperform recent SoTA models significantly by up to 7.1% *F*1. All the resources are publicly available (https://github.com/cpmss521/KGPC), which can facilitate the study of the few-shot BioNER.

## 2 Related work

### 2.1 Few-shot BioNER

Few-shot BioNER aims to locate and classify the entity based on only few labeled samples from each category. A series of approaches have been explored for few-shot named entity recognition. The method based on prompt learning is proposed to reduce the gap between training and fine-tuning (Chen *et al.* 2022b, Wang *et al.* 2022c). Instead of learning category prototype, Yang and Katiyar (2020) calculate the nearest neighbor of each sample in query set. Following this, Ma *et al.* (2022) introduce label information and measure the similarity between entity types and samples. However, most of these studies first train on the high-resource dataset (such as News doamin) and then transfer knowledge to the target low-resource dataset (biomedical domain). Cross-domain transfer learning inevitably brings domain shift problems.

### 2.2 Data augmentation

DA is an effective method to alleviate data scarcity in various natural language processing (NLP) tasks, such as text classification (Sun *et al.* 2021) or natural language understanding (Zhou *et al.* 2022a). However, it is challenging to extend DA method to token-level named entity recognition, such as token-label misalignment issue. Recent works (Ding *et al.* 2020, Zhou *et al.* 2022b) exploit pre-trained MLM to generate directly new synthetic data after fine-tuning for few-shot named entity recognition. Dai and Adel (2020) propose randomly substitute target entities with another entity of the same class. Unfortunately, the above DA method either generated unreliable entities or distort the original context.

### 2.3 Contrastive learning

Despite cross-entropy hitting the optimality in supervised learning, recent work shows its shortcomings, such as poor adversarial robustness (Pang *et al.* 2020). Drawing inspiration from the InfoNCE loss (van den Oord *et al.* 2018), many efforts on CL have been extensively explored, from vision to text, to learn high-quality representations. Among them, SimCLR (Chen *et al.* 2020), a simple CL framework, is proposed to learn the robustness of visual representations by constructing positive and negative pairs. Gao *et al.* (2021) extend CL to the NLP domain for sentence representations. Furtherly, Wu *et al.* (2022) add random Gaussian noise as an extension to the negative pairs based on prior work.

Although some studies have made progress in addressing certain challenges, the proposed KGPC superiors them in two aspects: (i) based on semantic relations of knowledge graph, we propose knowledge-guided instance generation for few-shot BioNER, which generates valid and diverse entities to augment samples and avoid cross-domain shift problem; (ii) formulating BioNER as a QA task, we propose prompt CL to better locate entity span by maximizing the MI of matching query–answer pairs while minimizing the MI of negative samples mismatching the query. Both efforts, jointly exploiting data and models together, are rarely studied to our best knowledge.

## 3 Materials and methods

**Figure 2.**
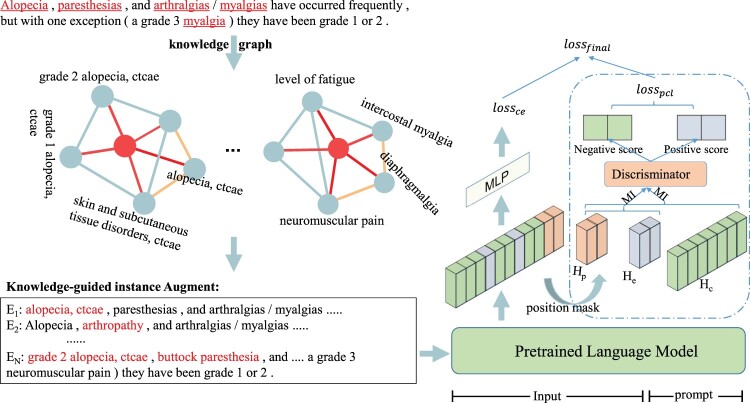
An overview of proposed KGPC for few-shot BioNER. (Left): with given few-shot samples, knowledge sub-graphs are constructed and then knowledge-guided instance generation products novel and diverse samples based on semantic relation of knowledge graph. (Right): Introducing question prompts, we natively formulate BioNER as a QA task and propose prompt CL by maximizing the MI of matching query–entity pairs and minimizing the MI of negative samples unmatched the query. Finally, the model is jointly optimized by cross-entropy loss and contrastive loss

Figure 2 presents the workflow of our proposed KGPC framework. Based on limited few-shot instances, we first generate new samples by knowledge-guided instances generation (Section 3.1). Then, introducing the query prompt, we convert BioNER into a QA task (Section 3.2) and maximize the MI of query–entity pairs by prompt CL (Section 3.3).

### 3.1 Knowledge-guided instances generation

Considering the biomedical knowledge graph contains abundant entities, we propose a knowledge-guided instance generation. The core idea is to replace the target entity with its neighbor nodes based on similar semantics relation. The method consists of two consecutive phases: knowledge sub-graph construction and entity replacement.

#### 3.1.1 Knowledge sub-graph construction

In the work, we utilize a domain knowledge base from the Unified Medical Language System (UMLS) (Bodenreider 2004), which contains Metathesaurus, Semantic Network, Specialist Lexicon, and Lexical Tools. Abundant fine-grained biomedical concepts and their relations are provided by Metathesaurus. And each concept is allocated one or more semantic types in the semantic network, such as “Receptor,” “Tissue” or “Organism.” In this article, to align with the prevailing literature on knowledge graphs, we refer to UMLS concepts as entities. Specifically, given the input sentence X $={\left\{x\right\}}_{i}^{n}$, we can collect all possible candidate entities by MetaMap (Aronson 2001), which is a UMLS mapping tool. We then construct a knowledge sub-graph K for each candidate entity, which lies in the central node. The relations and neighbor nodes are linked with the central node by looking up the Metathesaurus. It is worth noting that, in order to fit the textual context and preserve semantic consistency, we only consider six kinds of relations, i.e. “SY,” “RL,” “RB,” “RQ,” “PAR,” and “CHD.” The semantic types also served as type nodes that are associated with the central node when the candidate entity is not the entity mention. Finally, we denote knowledge sub-graph as $K={\left\{(h,r_{i},t_{i})\right\}}_{i}^{k}$, where *h* is central node and its neighbor node is $t_{i}$, $r_{i}$ and *k* represent a relation and the number of neighbor nodes, respectively.

#### 3.1.2 Entity replacement

During this phase, augmented instances are generated by replacing the central nodes *h* with their neighbors $t_{i}$. And the corresponding entity types remain unchanged, we just adjust BIO labels. For example, the B-Disease entity word “myalgias” is replaced by “neuromuscular pain” in B-Disease I-Disease. And we directly assign the label “O” to the neighbor node if the replaced candidate concept is not an entity. The number of generated instances is limited to *N* for each sentence. An example of knowledge-guided instance generation is in the left region of Fig. 2. Red color nodes represent candidate concepts (i.e. central nodes) from input text. The others denote the neighbor nodes.

### 3.2 Query prompt

Since query *Q* in question-answering tasks naturally provides hints for prompt construction, we formulate the BioNER problem as a QA task. Formally, given the question prompts $Q_{\mathrm{prompt}}$ and input sentence X, the task aims to extract the entity set $e=\{e_{1},\ldots ,e_{m}\}$. Using naive question prompts like “Can you detect [T] entity like $\left[E_{1}\right]$, $\left[E_{2}\right]$ ?” will simply work, where T is the set of pre-defined entity types, and $E_{1}$ and $E_{2}$ are biomedical entities. We can replace the [T] slot with an entity type and fill the $\left[E_{1}\right]$ or $\left[E_{2}\right]$ slots with the corresponding entities, which are selected randomly from the training dataset. Thus, we can obtain ||T|| question prompts for ||T|| entity types and ask questions from different perspectives for each sentence.

Then, we use pre-trained BioBERT (Lee *et al.* 2020) as the backbone model for QA. Given the question prompt $Q_{\mathrm{prompt}}$ and text sequence X, the input of the QA model is concatenated as: where CLS denotes a special token and SEP is a separator. Next, we obtain the contextual representation of the input sequence and question prompt, denoting as $H_{x}\in R^{n\times d}$ and $H_{p}\in R^{m\times d}$ respectively, where *n* and *m* are the length of sequence and prompt, *d* denotes the dimension of BioBERT. Considering that multiple answers might be contained in the input text, we feed the hidden state sequence $H_{x}$ into the softmax classification layer and predict sequence BIO probability. For each input text X $={\left\{x\right\}}_{i}^{n}$, the probability of sequence labels is calculated as: where *W* and *b* are trainable parameters. Consequently, we can extract entities from the label sequence by identifying the BIO boundaries.


(1) $$\left\{\left[\mathrm{CLS}],X,[\mathrm{SEP}],Q_{\mathrm{prompt}},[\mathrm{SEP}\right]\right\},$$

(2) $$\begin{array}{c} P_{X}(\theta )=\mathrm{softmax}\left(\mathrm{MLP}(W^{T}H_{x}+b)\right) \end{array},$$

### 3.3 Prompt CL

CL aims to learn a feature representation space that draws positive sample pairs together while pushing away negative sample pairs as much as possible. The main challenge of CL is that constructs positive and negative sample pairs. To better locate entity boundaries, we treat the matching question prompt and answer (i.e. entity) as positive pairs, otherwise as negative pairs. And then we compute the contrastive loss by maximizing the MI between latent feature space. In information theory, MI is a fundamental measure that quantifies the degree of dependence between query *Q* and answer *A*, i.e. the reduction of uncertainty in *A* given *Q*, which is formulated as: where H(A) is the information entropy of answer *A*, H(A|Q) denotes the uncertainty of *A* given query *Q*. Theoretically, assuming *Q* and *A* are completely related, then the uncertainty H(A|Q) = 0 and I(Q,A) is maximized. Inversely, if *Q* and *A* are independent of each other, H(A|Q)=H(A) and I(Q,A) is zero. Consequently, given questions, prompt CL can help the model precisely extract entities from the text.


(3) $$\begin{array}{c} I(Q,A)=H(A)-H(A|Q) \end{array},$$

The right region of Fig. 2 presents the prompt CL module. It provides fine-grained guidance to the query prompts to better answer matching entity mentions. Technically, based on sequence representation $H_{x}$ and question prompt $H_{p}$ in Section 3.2, we first obtain the entity mention matched with the question by position mask, which servers as positive samples. Meanwhile, those outside of the entity mentions in sequence are regarded as negative samples. Then, $H_{x}$ is divided into positive sample feature $\tilde{H_{e}}\in R^{e\times d}$ and negative sample feature $\tilde{H_{c}}\in R^{c\times d}$. Therefore, we can maximize the MI between the query $H_{p}$ and paired entity mentions $\tilde{H_{e}}$, as well as minimize the MI between the query $H_{p}$ and negative sample $\tilde{H_{c}}$. However, it is difficult for MI estimation to deal with high dimensional continuous random variables. In practice, an alternative way is to approximate MI by lower bound estimators (Hjelm *et al.* 2019, Dong *et al.* 2020). Therefore, we model the MI estimation as maximizing the value over the lower bound on MI with the Jensen–Shannon MI estimator (Hjelm *et al.* 2019) as: where sp refers to the softplus function with $\text{sp}(x)=\text{log}\;\left(1+e^{x}\right)$, and $T_{\theta }$ is a MI neural discriminator with trainable parameters θ. Note that only the second part is calculated during contrastive training when there is no entity in sentence. Finally, we get prompt contrastive loss $L_{\mathrm{pcl}}$ as follows:


(4) $$I_{\theta }^{c}=E_{\mathrm{pos}}[-\text{sp}\left(-T_{\theta }\left(H_{p},{\tilde{H}}_{e}\right)\right]-E_{\mathrm{neg}}\left[\text{sp}\left(T_{\theta }\left(H_{p},{\tilde{H}}_{c}\right)\right)\right],$$

(5) $$L_{\mathrm{pcl}}=-\text{argmax}\frac{1}{N}\left(\sum_{i=1}^{N}I_{\theta }^{c}\right).$$

### 3.4 Training objectives

To train the KGPC model, we joint optimize the training objective for few-shot BioNER by: where λ is a hyperparameter, $L_{\text{ce}}$ is cross-entropy loss, and $L_{\mathrm{pcl}}$ denotes the prompt contrastive loss over entity extraction.


(6) $$L_{\mathrm{final}}=\lambda L_{\text{ce}}+(1-\lambda )L_{\mathrm{pcl}},$$

## 4 Experiments

### 4.1 Datasets and evaluation

The model is evaluated on low-resource settings across NCBI, BC5CDR-Disease, BioNLP11EPI, and BioNLP13GE datasets. More details of the datasets are described as follows:

**NCBI** dataset (Doğan *et al.* 2014) originates from the named entity recognition and concept normalization, which consists of 793 PubMed abstracts annotated for the disease entity.

**BC5CDR-Disease** dataset (Li *et al.* 2015) contains 1500 PubMed articles with disease mentions. The corpus is divided into three sets of 500 PubMed articles each, dedicated to training, developing, and testing the model.

**BioNLP** dataset, including BioNLP11EPI (Kim *et al.* 2011) and BioNLP3GE dataset (Nédellec *et al.* 2013), comes from the Biomedical Natural Language Processing Workshops. The basic entities contain gene or protein.

To evaluate few-shot performance on BioNER task, the precision (*P*), recall (*R*), and micro-averaged *F*1 score are used in our experiment.

### 4.2 Low-resource setting

Following Ma *et al.* (2022), we downsample (at sentence level) the original training set in each dataset to construct a *K*-shot support set. It means that each entity type contains *K* samples in support set. Specifically, we sample 5, 20, and 50 sentences (at least once for each entity type) for all datasets to simulate the low-resource scenario. The overview statistic of the few-shot data sampling on BioNER is shown in Table 1. In prior works (Athiwaratkun *et al.* 2020, Hou *et al.* 2020), test set is also downsampled to construct *K*-shot query set. Unlike them, we directly evaluate the model in the full test set, which confirms to real-world cases. To avoid the influence of random sampling, we repeatedly downsample five times for each few-shot setting with different random seeds and report the average *F*1 score with standard deviation.

**Table 1.** Number of sentences in few-shot data for BioNER.

Dataset	Support set			Dev	Test
	5 shot	20 shot	50 shot		
NCBI	5	20	50	923	940
BC5CDR-disease	5	20	50	4581	4797
BioNLP11EPI	5	20	50	1955	4122
BioNLP13GE	5	20	50	2737	3391

### 4.3 Training details

We adopt BioBERT-base cased version as the backbone transformer model. For all four benchmark datasets: the number of generated instances *N* is set to nine at most according to the support set; the training epoch number is 20; the hyperparameter λ is set to 0.5; Adamw optimizer (Loshchilov and Hutter 2019) with a warmup-decay schedule is applied to train our model. In implementing TransferBERT, following Chen *et al.* (2022b), we regard the CoNLL-2003 as the high-resource domain dataset, which is pre-fine-tuned on 10 epochs and continues fine-tuning on low-resource datasets. The experiments are implemented under the PyTorch framework and trained on NVidia RTX A5000 GPU.

### 4.4 Compared methods

In the experiments, we compare our method with several recent SoTA FSL models on BioNER task:

**TransferBERT** is a domain transfer model performing sequence labeling following Devlin *et al.* (2019), which is first pre-fine-tuning on high-resource domains and then further fine-tune on the low-resource domain.

**Daga** (Ding *et al.* 2020) proposes a DA method that linearizes labeled sentences, and then uses them to train a language model to learn the distribution of tokens and labels for generating synthetic training data.

**NNshot** (Yang and Katiyar 2020) trains the nearest neighbor classifier based on instances in the source domain for few-shot named entity recognition. We alternatively utilize the CONLL03 dataset due to the source dataset is unavailable in the original paper, which is consistent with the LightNER method (Chen *et al.* 2022b).

**LightNER** (Chen *et al.* 2022b) formulates sequence labeling as generative framework, which generates entity spans and types by incorporating continuous prompts into the attention layer to adapt pre-trained BART-large (Lewis *et al.* 2020) model weights. And the model is fine-tuned by high-resource datasets to transfer knowledge for low-resource few-shot named entity recognition in general domain.

**FFF-NER** (Wang *et al.* 2022c) formulates the named entity recognition as masked token prediction and generation by prompt-tuning the pre-trained model. And two tokens, “is-entity” and “which-type,” are introduced for span detection and type prediction.

**MELM** (Zhou *et al.* 2022b) aims to generate augmented data by masked entity language modeling for FSL in the general domain. They first insert the entity label into the input sentence and randomly mask the entity tokens, and then fine-tune the language model, like XLM-RoBERTa-Large (Conneau *et al.* 2020), to predict masked entity tokens by explicitly conditioning on their labels.

We reproduce their methods in our experimental environment. Note that we replace their encoders with BioBert except LightNER and MELM. The augmented instances, in MELM and Daga models, keep the same size as ours. In addition, all parameter settings remain consistent with reported in their paper for a fair comparison.

## 5 Main results

Table 2 summarizes the experiment results under 5-shot, 20-shot, and 50-shot in four benchmark datasets. From the results, it can be seen that (i) the proposed KGPC achieves the best performance in almost all settings, except on the 50-shot setting where the BC5CDR-disease dataset is sampled. Notably, our model performs significantly better than previous SoTA methods, with an average increase of 9.2%, 7.1%, and 8.4% in *F*1 score across the three few-shot settings on the NCBI dataset, respectively. (ii) Compared with DA method based on MLM, the proposed knowledge-guided data generation achieves up to 19.8%, 13.3%, 8.1%, and 7.7% improvement over baseline models (Ding *et al.* 2020, Zhou *et al.* 2022b) on NCBI, BC5CDR-Disease, BioNLP11EPI, and BioNLP13GE, respectively. It demonstrates that knowledge-guide instance generation is more effective than the MLM-based method, which generates invalid entities and meaningless augmented samples. (iii) Vanilla cross-domain transfer learning methods, like TransferBERT and NNshot, show the poor ability of FSL over LightNER and FFF-NER, which demonstrates prompt-tuning can reduce the gap between pre-training and fine-tuning. And it may be the reason why our model is slightly inferior to theirs under the 50-shot setting on the BC5CDR-disease dataset. (iv) Among all baselines across four datasets, the prompt learning-based method (such as LightNER and FFF-NER) presents a competitive baseline that leverages entity type prompt information, yet our method can obtain gains from two perspective: data and model. Firstly, by data augment with knowledge-guided instance generation, the proposed KGPC method can alleviate data sparsity and avoid over-fitting of the model. Secondly, introducing question prompts, we formulate BioNER as a QA task and propose prompt CL to improve the robustness of the model.

**Table 2.** Performance comparison with other SoTA methods on all datasets.^a^

Dataset	Model	5 shot			20 shot			50 shot		
		*P* (%)	*R* (%)	*F*1 (%)	*P* (%)	*R* (%)	*F*1 (%)	*P* (%)	*R* (%)	*F*1 (%)
NCBI	TransferBERT (Devlin *et al.* 2019)	28.7 ± 3.3	30.5 ± 13.7	27.6 ± 6.3	46.6 ± 7.2	57.0 ± 12.2	49.5 ± 3.6	48.7 ± 4.9	67.5 ± 6.5	56.2 ± 3.6
	Daga (Ding *et al.* 2020)	35.7 ± 11.1	19.3 ± 13.6	20.7 ± 8.3	43.5 ± 6.6	56.2 ± 8.6	48.7 ± 6.4	44.6 ± 2.1	64.8 ± 6.1	52.6 ± 1.9
	NNshot (Yang and Katiyar 2020)	17.4 ± 3.0	37.1 ± 9.8	23.2 ± 3.8	23.9 ± 3.8	47.3 ± 7.5	31.7 ± 4.7	26.9 ± 3.3	51.5 ± 5.2	35.2 ± 3.3
	LightNER (Chen *et al.* 2022b)	43.7 ± 4.1	41.9 ± 7.5	42.4 ± 4.7	54.6 ± 4.4	59.0 ± 4.6	56.4 ± 1.9	60.53 ± 6.0	61.9 ± 5.1	60.9 ± 3.7
	FFF-NER (Wang *et al.* 2022c)	35.7 ± 10.2	24.9 ± 6.5	29.2 ± 7.8	45.7 ± 9.7	54.1 ± 10.7	47.5 ± 2.7	55.5 ± 6.2	74.5 ± 6.2	63.3 ± 4.8
	MELM (Zhou *et al.* 2022b)	35.4 ± 5.3	30.8 ± 6.8	31.8 ± 1.6	36.5 ± 1.7	**71.2 ** ± 2.1	48.2 ± 1.4	53.9 ± 4.7	67.4 ± 5.2	59.5 ± 1.8
	KGPC (ours)	**52.2 ** ± 2.2	**52.1 ** ± 9.6	**51.6 ** ± 5.0	**59.4 ** ± 5.7	68.8 ± 7.1	**63.5 ** ± 4.8	**68.3 ** ± 1.4	**75.6 ** ± 4.6	**71.7** ± 2.3

BC5CDR-disease	TransferBERT (Devlin *et al.* 2019)	24.7 ± 8.2	13.0 ± 6.3	16.4 ± 5.6	40.3 ± 6.2	51.7 ± 7.9	44.6 ± 3.6	48.6 ± 4.0	65.3 ± 2.6	55.5±2.0
	Daga (Ding *et al.* 2020)	34.4 ± 6.8	19.4 ± 4.1	24.2 ± 3.1	37.4 ± 4.9	53.2 ± 6.3	43.4 ± 2.1	47.2 ± 5.4	63.0 ± 3.5	53.6 ± 2.0
	NNshot (Yang and Katiyar 2020)	18.5 ± 4.9	37.2 ± 5.7	34.2 ± 4.5	20.1 ± 4.9	43.0 ± 6.7	26.9 ± 4.3	22.4 ± 2.3	47.3 ± 3.8	30.4 ± 2.7
	LightNER (Chen *et al.* 2022b)	39.8 ± 8.6	33.2 ± 12.3	35.4 ± 9.7	**58.3 ** ± 4.1	54.1 ± 6.2	55.7 ± 2.2	63.0 ± 4.3	66.0 ± 1.1	64.4 ± 2.6
	FFF-NER (Wang *et al.* 2022c)	43.6 ± 10.2	44.6 ± 5.8	43.1 ± 4.4	42.9 ± 14.2	53.7 ± 8.2	45.1 ± 5.6	**66.6 ** ± 7.7	**71.4 ** ± 7.3	**68.2 ** ± 1.8
	MELM (Zhou *et al.* 2022b)	34.2 ± 2.2	41.3 ± 6.7	37.0 ± 2.3	39.5 ± 5.9	60.3 ± 3.5	47.5 ± 4.8	51.9 ± 3.4	65.2 ± 5.5	57.6 ± 3.0
	KGPC (ours)	**50.8 ** ± 9.8	**51.3 ** ± 7.4	**50.4 ** ± 7.2	56.6 ± 1.5	**65.2 ** ± 4.1	**60.5 ** ± 2.4	59.0 ± 2.6	67.8 ± 3.0	63.0 ± 1.9

BioNLP11EPI	TransferBERT (Devlin *et al.* 2019)	41.9 ± 0.6	61.8 ± 4.8	49.8 ± 1.1	46.7 ± 2.4	65.1 ± 7.0	54.1 ± 1.8	50.7 ± 2.0	73.6 ± 0.7	60.0 ± 1.1
	Daga (Ding *et al.* 2020)	44.7 ± 6.9	48.0 ± 18.8	44.8 ± 13.5	47.7 ± 1.4	68.6 ± 4.1	56.2 ± 1.5	51.4 ± 3.3	74.8 ± 3.7	60.8 ± 1.4
	NNshot (Yang and Katiyar 2020)	31.1 ± 7.6	42.3 ± 14.4	35.6 ± 10.4	32.2 ± 1.2	47.8 ± 3.1	38.4 ± 1.4	36.5 ± 1.3	56.0 ± 4.0	44.2 ± 2.1
	LightNER (Chen *et al.* 2022b)	**46.0 ** ± 1.6	45.4 ± 12.1	44.6 ± 7.4	53.1 ± 1.4	56.2 ± 2.4	54.6 ± 1.1	61.3 ± 2.4	61.0 ± 5.9	60.9 ± 2.0
	FFF-NER (Wang *et al.* 2022c)	45.1 ± 6.8	53.2 ± 11.2	47.2 ± 3.1	47.8 ± 11.1	57.1 ± 9.9	50.1 ± 1.8	53.5 ± 3.4	75.4 ± 5.8	62.3 ± 2.5
	MELM (Zhou *et al.* 2022b)	38.1 ± 4.6	62.1 ± 11.6	46.4 ± 3.9	44.0 ± 3.7	67.9 ± 4.3	53.1 ± 1.7	48.7 ± 1.7	74.7 ± 2.5	58.9 ± 1.9
	KGPC (ours)	45.8 ± 13.5	**61.9 ** ± 3.6	**51.4 ** ± 10.8	**55.3 ** ± 3.0	**69.5 ** ± 2.9	**61.4 ** ± 1.5	**61.7 ** ± 2.2	**78.0 ** ± 1.2	**68.9 ** ± 1.5

BioNLP13GE	TransferBERT (Devlin *et al.* 2019)	34.9 ± 5.6	42.8 ± 13.6	37.7 ± 8.3	48.5 ± 3.7	66.0 ± 5.9	55.6 ± 1.7	52.0 ± 3.7	73.9 ± 2.7	60.9 ± 1.9
	Daga (Ding *et al.* 2020)	44.0 ± 11.5	38.0 ± 6.6	39.6 ± 3.9	55.4 ± 4.3	71.5 ± 3.1	62.2 ± 1.7	53.1 ± 3.3	74.7 ± 2.1	62.0 ± 2.4
	NNshot (Yang and Katiyar 2020)	26.5 ± 6.7	33.5 ± 6.2	29.1 ± 5.1	37.1 ± 3.1	52.7 ± 4.7	43.4 ± 2.7	38.4 ± 1.1	57.0 ± 2.7	45.9 ± 1.6
	LightNER (Chen *et al.* 2022b)	37.6 ± 5.0	44.8 ± 4.1	40.8 ± 3.9	56.2 ± 3.8	59.2 ± 8.0	57.5 ± 5.2	58.3 ± 2.2	68.5 ± 2.1	63.0 ± 1.5
	FFF-NER (Wang *et al.* 2022c)	**47.9 ** ± 4.0	42.8 ± 4.8	44.8 ± 1.0	48.8 ± 5.6	65.8 ± 7.6	55.5 ± 3.8	57.0 ± 5.4	76.3 ± 4.9	65.1 ± 4.0
	MELM (Zhou *et al.* 2022b)	33.6 ± 7.6	**54.9 ** ± 6.3	41.2 ± 6.6	47.8 ± 3.2	76.7 ± 2.8	58.8 ± 2.9	46.5 ± 2.6	70.2 ± 6.0	55.8 ± 2.7
	KGPC (ours)	40.9 ± 7.2	52.4 ± 11.2	**45.3 ** ± 7.6	**61.7 ** ± 1.1	**71.3 ** ± 3.4	**66.1 ** ± 1.7	**63.6 ** ± 1.6	**77.2 ** ± 2.8	**69.7 ** ± 1.4

a The results are evaluated on full test sets and reported the average of five runs with different support set sampling. The bold values denote the highest scores and the second best are wave-lines.

## 6 Discussion

### 6.1 Impact of the DA

**Figure 3.**
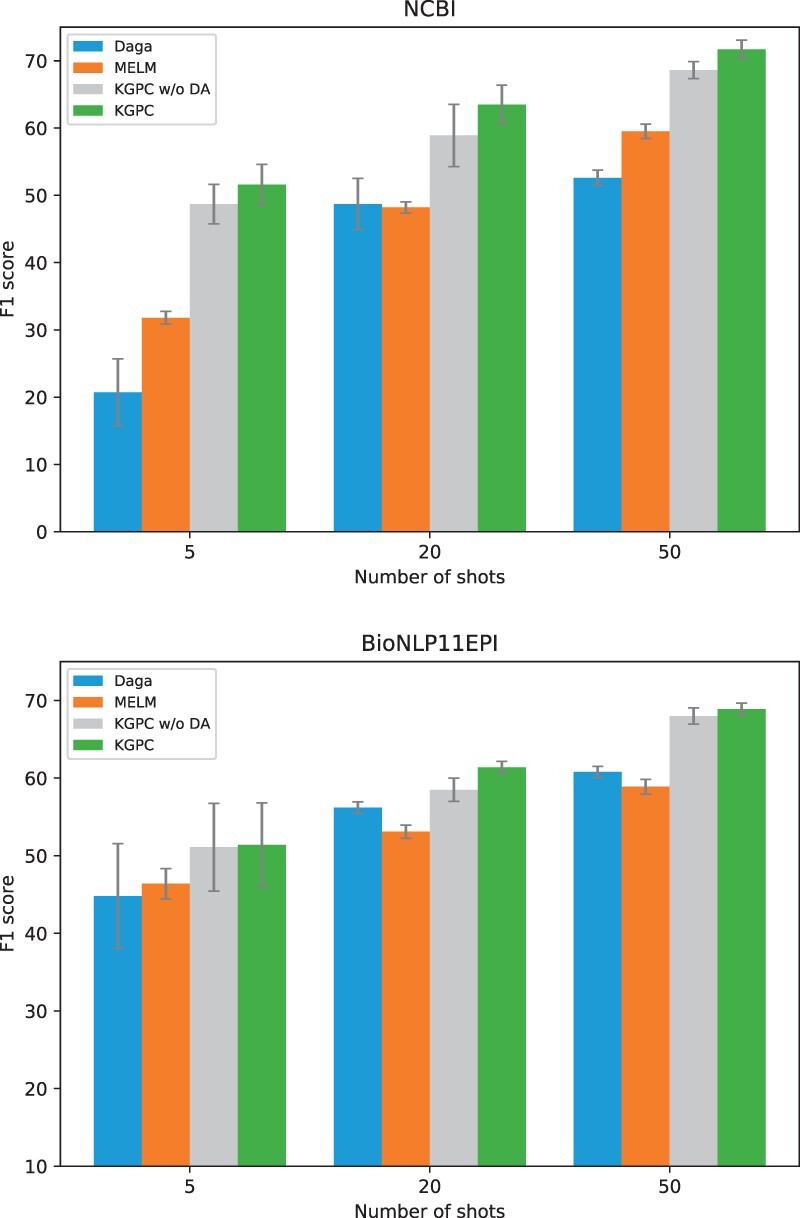
Effect of DA on the NCBI and BioNLP11EPI dataset

To assess the efficacy of the proposed knowledge-guided instance generation (Section 3.1), a series of ablation studies are conducted on NCBI and BioNLP11EPI datasets. As shown in Fig. 3, we directly train the model on support set without DA, denoted as KGPC w/o DA. We observe an obvious performance drop compared with KGPC, which demonstrates leveraging knowledge semantic relations indeed helps KGPC generates valid and diverse entities, and endow better generalization. Meanwhile, KGPC w/o DA also shows its superior performance compared to the recent SoTA DA method (Ding *et al.* 2020, Zhou *et al.* 2022b) with up to 16.9% and 4.7% absolute improvement under five-shot setting in NCBI and BioNLP datasets, respectively. We attribute it to the fact that prompt CL enhances the quality of the entity representations, thus resulting in improved generalization.

### 6.2 Data fidelity check

**Figure 4.**
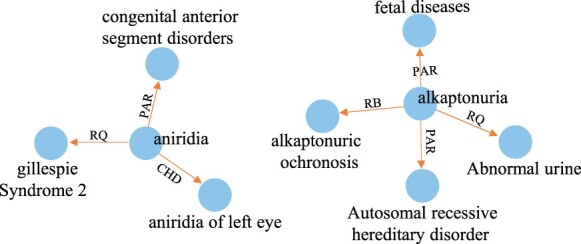
Knowledge semantic relation graph

In addition, by comparing the instances generated from MELM (Zhou *et al.* 2022b) and our KGPC, we further perform the data fidelity check. As shown in Table 3, we can see that the tokens generated by MELM (Zhou *et al.* 2022b) are extremely similar to the replaced entity and it does not exist in the real world. In other words, it produces virtual and task-unrelated data. We attribute it to the sub-word segmentation technology, like Byte Pair Encoding, used in the pre-training process of the language model. In contrast, our KGPC controls to generate valid entities by semantic relation of knowledge graph. Specifically, by looking at the parent (i.e. PAR) and child (i.e. CHD) relations of the entity mention “aniridi,” we can obtain neighbor nodes “congenital anterior segment disorders” and “aniridia of left eye” as shown in Fig. 4 (we present partial relation for brevity). Different from generative augmentation by MLM (e.g. MELM and Daga), our knowledge-guided instance generation is more interpretable based on knowledge semantics relations. Furthermore, it is noteworthy that generated augmentation samples by our KGPC not only fit the original context semantics but also spawn more diverse entities.

**Table 3.** Data fidelity check.^a^

E1: the frequency at which we have found PAX6 mutations suggests that lesions in PAX6 will account for most cases of ${\left[\mathrm{aniridia}\right]}_{\text{Disease}}$.	
MELM	anisodia, aniride, aniriasis, anisoemia, aniseal, ⋯
KGPC	gillespie syndrome 2, aniridia and absent patella syndrome, congenital anterior segment disorders, aniridia of left eye, ⋯

E2: we were able to map the human gene to chromosome 3q in six ${\left[\mathrm{alkaptonuria}\right]}_{\text{Disease}}$ pedigrees of Slovak origin.	
MELM	alvetonuria, alcetonin, alkapilliuria, osteokaptonuria, ⋯
KGPC	alkaptonuric ochronosis, fetal diseases, abnormal urine, autosomal recessive hereditary disorder, ⋯

a The underlined words indicates words that do not match the given label.

### 6.3 Impact of prompt CL

Previous research (Zhao *et al.* 2021) has shown that different prompt mechanisms may lead to various performances. To investigate such influence, we conduct experiments with two prompt strategies on the 20-shot setting across four benchmark datasets. The central idea of the label conditional prompt is utilizing entity types, with the form of “The [T].” The slot is filled with an entity type from label sets. The question-style prompt is a query with “can you detect [T] entity like [$E_{1}$], [$E_{2}$] ?,” which is in line with human intuition. Table 4 reports the results of two prompt strategies. The results show that prompts with question-style work the best in general. Therefore, CL is performed under the query prompting strategy in our experiment.

**Table 4.** The effects of prompt strategy on different datasets.

Dataset	Label conditional prompt			Query prompt		
	*P* (%)	*R* (%)	*F*1 (%)	*P* (%)	*R* (%)	*F*1 (%)
NCBI	58.3	56.9	57.6	59.4	68.8	63.5
BC5CDR-disease	54.0	63.7	58.4	56.6	65.2	60.5
BioNLP11EPI	48.3	65.3	55.6	55.3	69.5	61.4
BioNLP13GE	56.9	76.1	65.1	61.7	71.3	66.1

**Figure 5.**
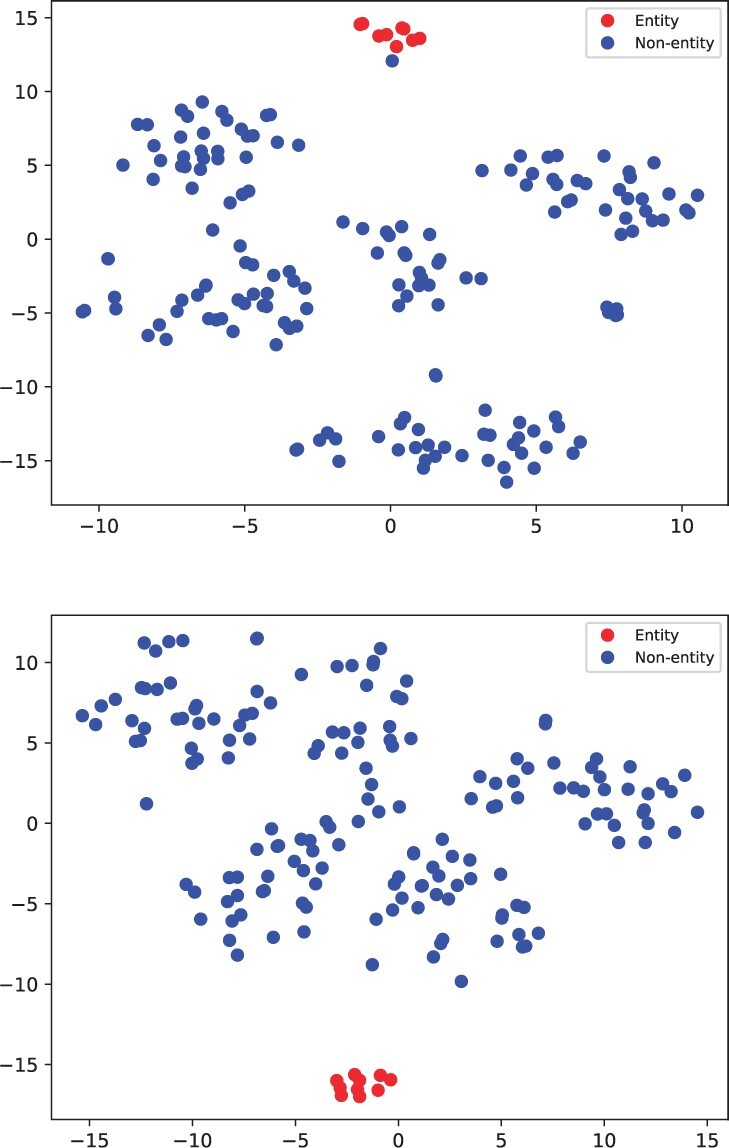
Visualization of the NCBI test data representations using *t*-SNE for KGPC w/o CL (upper) and KGPC with CL (lower)

In Table 5, we study the effect of CL under different FSL settings on two benchmark datasets. We directly remove CL module during training, denotes as KGPC w/o CL. We observe that, on average, the performance without CL is significantly inferior to KGPC, particularly under the extremely low-resource scenario (i.e. five-shot), dropping 10.2% and 4.6% on the NCBI and BioNLP11EPI dataset. Recall that prompt CL adopts MI estimation to enlarge the similarities of matching query–answer (entity mentions) pairs, and reduce similarities between unpaired queries and entity mentions; this is in line with the question-answering objective. Thus, confusion from other redundant information (i.e. non-entity) is avoided, KGPC can precisely extract the correct entity answers. In this case, CL always keeps different degrees of benefit on two datasets. Furthermore, as shown in Fig. 5, we conduct the *t*-SNE visualization experiments of the learned representations of five random instances on the NCBI test set, comparing KGPC with and without the CL. It clearly reveals that prompt CL enforces entities that match with the query more compact clustering.

**Table 5.** The effects of CL on different datasets.

Dataset	Shot	KGPC			KGPC w/o CL		
		*P* (%)	*R* (%)	*F*1 (%)	*P* (%)	*R* (%)	*F*1 (%)
NCBI	5	52.2	52.1	51.6	42.8	40.0	41.4
	20	59.4	68.8	63.5	62.8	62.0	62.4
	50	68.3	75.6	71.7	66.4	66.0	66.2

BioNLP11EPI	5	45.8	61.9	51.4	46.5	47.1	46.8
	20	55.3	69.5	61.4	65.2	55.1	59.6
	50	61.7	78.0	68.9	59.4	75.9	66.7

### 6.4 Case study

Finally, to evaluate the advantages of proposed KGPC model, we randomly sample some test samples predicted by different models on the NCBI dataset, as shown in Table 6. With the aid of knowledge-guided instance generation, our KGPC model successfully identifies the entire Disease entity in Case 1. Conversely, the baseline models all identify it as two separate entities due to the effect of punctuation. In Case 2, the KGPC model without DA incorrectly predicts “Bedlington” as a Disease entity. In contrast, our KGPC model with augmented samples could avoid over-fitting during training, thus correctly inferring Disease entity. These demonstrate that the proposed knowledge-guided instance generation is more reliable than masked entity language modeling (i.e. MELM model) and can improve the performance of the few-shot BioNER task.

**Table 6.** Case study of the prediction results on BC5CDR test dataset.

Case 1:	
True label	Risk of developing ${\left[\mathrm{colorectal},\mathrm{breast and other cancers}\right]}_{\text{Disease}}$ were compared between genotyped ⋯
KGPC	${\left[\mathrm{colorectal},\mathrm{breast and other cancers}\right]}_{\text{Disease}}$
KGPC w/o DA	${\left[\mathrm{colorectal}\right]}_{\text{Disease}}$ ${\left[\mathrm{breast and other cancers}\right]}_{\text{Disease}}$
MELM	${\left[\mathrm{colorectal}\right]}_{\text{Disease}}$ ${\left[\mathrm{breast and other cancers}\right]}_{\text{Disease}}$

Case 2:	

True label	Genetic mapping of the ${\left[\mathrm{copper toxicosis}\right]}_{\text{Disease}}$ locus in Bedlington terriers to dog chromosome ⋯
KGPC	${\left[\mathrm{copper toxicosis}\right]}_{\text{Disease}}$
KGPC w/o DA	${\left[\mathrm{copper toxicosis}\right]}_{\text{Disease}}$ ${\left[\mathrm{Bedlington}\right]}_{\text{Disease}}$
MELM	${\left[\mathrm{copper toxicosis}\right]}_{\text{Disease}}$

## 7 Conclusion

Considering a more realistic scenario, in this work, we propose a KGPC framework for few-shot BioNER, which makes it adapt to low-resource scenarios promptly from data and model structure two perspectives. To overcome the data sparsity of low-resource, we proposed knowledge-guided instance generation, which generates valid and novel entity mentions by semantic relations of the knowledge graph. And by introducing question prompts, we natively formulate BioNER as a QA task, and propose prompt CL to improve the robustness of the model by measuring the ML between query and entity. The results demonstrate that proposed method is consistently superior to recent SoTA models. In the future, we will extend the proposed DA method based on knowledge semantics relation to other related tasks, such as few-shot biomedical relation extraction.

## Data Availability

The KGPC data underlying this article are available at https://github.com/cpmss521/KGPC.
